# Emergency department utilisation by patients with a diagnosis of borderline personality disorder: An acute response to a chronic disorder

**DOI:** 10.1111/1742-6723.13970

**Published:** 2022-03-30

**Authors:** Jillian H Broadbear, Joe‐Anthony Rotella, Donna Lorenze, Sathya Rao

**Affiliations:** ^1^ Spectrum Personality Disorder Service Melbourne Victoria Australia; ^2^ Faculty of Medicine, Nursing and Health Sciences Monash University Melbourne Victoria Australia; ^3^ Department of Emergency Medicine Northern Health Melbourne Victoria Australia; ^4^ School of Nursing, Midwifery and Social Sciences Central Queensland University Mackay Queensland Australia

**Keywords:** audit, borderline personality disorder, emergency department, self‐injury, suicidality

## Abstract

**Objective:**

Patients with borderline personality disorder (BPD) are likely to attend the ED while experiencing crises and associated self‐injury and suicidality. Our study describes the prevalence, features, and outcomes associated with ED presentations by patients diagnosed with BPD in Outer Eastern Melbourne, Australia.

**Methods:**

A retrospective electronic audit of 157 364 ED attendances identified 700 unique BPD‐related ED presentations between May 2015 and April 2016. For the purpose of comparison, 583 (81% female) of these 700 cases were matched with ‘depression only’ cases. ED re‐presentation data were also extracted.

**Results:**

The 583 matched BPD patients attended ED a total of 2807 times during the audit year compared with 1092 attendances for matched depression‐only patients. BPD patients were more likely to: arrive by ambulance (50%); have comorbid substance abuse (44%); have a psychotic (15%) or bipolar disorder (17%); be under the care of a psychiatrist (31%); be case‐managed (42%); and be admitted to an inpatient unit (21%). ED doctors saw 38% of BPD or depression patients within the recommended time according to their triage category. The majority (73%) of BPD patients attended ED more than once during the audit year (average 4.81 ± 6.63 times; range 2–78).

**Conclusion:**

Repeated ED attendance of a subset of patients diagnosed with BPD highlights both the severity of their presentation and the inadequacy of community mental health services for meeting their complex needs. Development of effective ED referral pathways with follow‐up to engage patients in BPD‐appropriate treatment will reduce the likelihood of crises and reliance on hospital EDs for acute episodic care.

**Key findings**

- People diagnosed with BPD who present to ED are experiencing severe and complex mental health challenges.
- These patients are likely to re‐attend ED, suggesting a lack of access to community BPD‐specific treatment options.
- Patients with BPD accounted for only 1.8% of all ED attendances despite their reputation for high emergency service utilisation.

## Introduction

People diagnosed with borderline personality disorder (BPD) experience difficulties with emotion regulation, impulsivity, relationship stability and self‐esteem.^1^ Crises associated with BPD entail intense emotional distress or its consequences; chronic suicidality is a frequent element of the diagnosis.^2^ Australian estimates suggest that 26% of emergency mental health presentations and 25% of inpatient mental health admissions are for patients meeting ICD‐10 criteria for personality disorder and related conditions.^3^

People with BPD come to ED following failed attempts at sourcing support in the community or consequent to maladaptive self‐management strategies including self‐injurious behaviour.^4^ Co‐occurring depression, anxiety, substance use and eating disorders may also prompt ED presentations.^5^ Although the prevalence of BPD in Australia is around 1%,^6^ as many as 10% of people whose lives end in suicide have this diagnosis.^7^, ^8^ Australian research shows that 25% of people diagnosed with BPD who die by suicide attended an ED within 6 weeks of their death.^7^

BPD is likely the most stigmatised mental health disorder, even among mental health professionals.^9^, ^10^ Stigma associated with BPD and difficulties staff experience when caring for patients with BPD may make the ED a counterproductive environment, contributing to iatrogenic harm.^11^

There is a paucity of research describing the experience of BPD patients attending the ED.^12^ Given that people with a BPD diagnosis may present frequently to ED,^13^, ^14^ there is clearly more to be learned about this population and understanding how to best meet their needs.

The aim of the present study was to describe the prevalence and nature of ED presentations by patients previously diagnosed with BPD. The BPD patient cohort was compared with depression‐only patients to highlight quantifiable differences in ED experience and management. Improving our understanding in this way will highlight the need for improved screening for BPD in ED, brief psychological intervention training for staff and the development of effective referral strategies to reduce crisis‐driven ED presentations of people suffering from BPD.

## Methods

A retrospective electronic audit of ED presentations was conducted at three university‐affiliated hospitals in Melbourne, Australia, that collectively managed 157 364 ED presentations between 1 May 2015 and 30 April 2016. Institutional Human Research Ethics Committee approval was granted (LR64‐2016).

The inclusion criteria were as follows:

- Patients with a confirmed diagnosis of BPD presenting to ED for a mental health‐related reason; or
- Patients with a confirmed diagnosis of depressive disorder but without an actual or suspected diagnosis of BPD, matched with respect to age, sex, the mental health‐related reason for presentation to ED (comparator group).

The electronic audit was conducted using Symphony (EMIS Health, Liverpool, England). Search terms used to identify the BPD cohort included Borderline Personality Disorder, BPD, Personality Disorder AND (self‐harm, suicide attempt, suic*, overdose, poisoning, superficial injury, wound, behavio?*). Search terms used to identify the depression cohort included Depression, MDD, Major Depressive Disorder AND (self‐harm, suicide attempt, suic*, overdose, poisoning, superficial injury, wound, behavio?*). The depression audit identified 11 761 cases; the BPD audit 2807 cases. Unique attendances following removal of re‐attendances were 8304 for depression and 700 for BPD. Only cases with a definitive diagnosis of BPD were included in the latter group.

Microsoft Access™ was used to code the patient files. De‐identified data collection included: age, sex, hospital site, reason for presentation (self‐harm, thought disorder, social, behavioural, suicidal ideation, overdose), triage category, attendance dates and times, time elapsed before being seen by emergency and mental health clinicians, additional service involvement (e.g. psychiatrist, GP, case manager), diagnoses and co‐morbidities including drug misuse, forensic and social data if available, referral after discharge and frequency of ED presentations (within 1 week, past month, the audit period, and total attendances over a 5–6 year period). Hospital records used to determine longer‐term ED presentation frequency dated back to April 2010 or March 2011 (hospital dependent).

### Data analysis

The BPD presentations were coded first; depression files were individually matched to BPD files based on age, sex and reason for presentation. The number of matched cases available for subsequent analysis was 583. χ^2^ tests were used to determine whether there were statistically significant differences in the frequencies with which the two cohorts exhibited characteristics of interest.

## Results

The 583 matched patients diagnosed previously with BPD (81% female) attended the ED a total of 2807 times for a mental health‐related reason during the 12‐month audit period, representing 1.8% of all ED attendances across the three hospitals. The majority of presentations were for suicidal ideation, self‐injury or overdose. The majority (71.6%) of patients were aged less than 40 years; 8.1% were 12–17 years, 31.9% were 18–25 years and 31.6% were 26–39 years. Of the remaining patients, 27.3% were aged between 40 and 60 years, with 1.2% aged over 60. The distribution of triage categories (and maximum recommended time to receive treatment) assigned to patients with BPD on presentation to ED were as follows: category 1, 2.2% (2 min); category 2, 14.3% (10 min); category 3, 57.1% (30 min); category 4, 25.3% (60 min) and category 5, 1.0% (2 h).

Patients with BPD were more likely to have co‐occurring mental health disorders such as substance use disorder, schizophrenia, bipolar disorder and/or eating disorder in comparison with patients in the ‘depression only’ cohort (Table 1). Patients diagnosed with BPD were more likely to have been transported to ED by ambulance (50% *vs* 36%) whereas ‘depression only’ patients were more likely to arrive by car (57% *vs* 43%).

**TABLE 1:** Co‐occurring disorders in ED patients diagnosed with BPD or depression‐only

Feature/comorbidity	% BPD cases	% Depression cases
BPD	100	0.0
Other personality disorder	9.6	0.0
Substance use disorder	43.9*	31.4
Depression	66.7	100
Schizophrenia/psychosis	14.8*	3.9
Anxiety	50.6	47.0
Bipolar affective disorder	16.6*	6.9
Eating disorder	7.5**	4.3

* *P* < 0.001, χ^2^ analysis.

** *P* < 0.05.

BPD, borderline personality disorder.

Community mental health supports differed for the two patient cohorts, with BPD patients significantly more likely to be case managed (reserved for people diagnosed with severe and complex mental health disorders) and under the care of a psychiatrist (Table 2).

**TABLE 2:** Community mental health supports for patients attending ED with BPD or depression

Mental health provider	BPD cases no. (%)	Depression cases no. (%)
Psychiatrist	180 (30.9)*	75 (12.9)
Psychologist	133 (22.8)	125 (21.4)
Case manager	242 (41.5)*	41 (7.0)
General practitioner	426 (73.1)	507 (87.0)*

* *P* < 0.001, χ^2^ analysis.

BPD, borderline personality disorder.

Patients with BPD whose primary reason for ED presentation was drug overdose (*n* = 133; 22.8%), were most likely to have overdosed on prescription medications (*n* = 116; 87%). Paracetamol (acetaminophen) overdose was a factor in 33 BPD patient presentations (5.7%). Secondary alcohol intoxication was noted in 37 BPD patients (6.3%).

With respect to referral from ED, BPD patients were more likely to be admitted to a mental health inpatient unit, to community mental health services, or have a medical admission (Table 3). Despite the greater involvement of psychiatrists in the care of patients with BPD (180 patients), only 21 were referred to a psychiatrist.

**TABLE 3:** Referral pathways from ED for patients with BPD or depression diagnoses

Category	BPD cases	% BPD	Depression cases	% Depression
General practitioner	149	25.56	216*	37.05
Inpatient unit	122*	20.93	78	13.38
Community Mental Health Services	117**	20.07	79	13.55
Medical admission	58**	9.95	30	5.15
Acute outpatient service	25	4.29	52**	8.92
Medical specialist	15	2.57	36**	6.17
Psychiatrist	21	3.6	26	4.46
Intensive care unit	9	1.54	6	1.03
Custodial	7	1.20	4	0.69
AOD service	6	1.03	11	1.89
Psychologist	7	1.2	16	2.74
NGO: residential	6	1.03	0	0
NGO: community	3	0.51	3	0.51
No referral	29	4.97	21	3.6

* *P* < 0.001, χ^2^ analysis.

** *P* < 0.01.

AOD, alcohol and other drug; BPD, borderline personality disorder; NGO, non‐government organisation.

In comparison with all national ED attendances reported to the Australian Institute of Health and Welfare,^15^ patients in the BPD‐diagnosed cohort were more likely to be female, younger in age and arrive by ambulance (Table 4). Patients from the BPD and depression‐only cohorts were less likely to be treated within the recommended timeframe according to their triage category.

**TABLE 4:** Comparison of the BPD‐diagnosed patient cohort characteristics with nationally reported figures for all ED presentations (AIHW annual report for public hospital system 2015–2016)

Endpoint	AIHW report	Our study
Male presentations	51%*	19%
Age at time of ED presentation (%)†	15–34 (27.5)*	18–39 (63.5)
Arrival by ambulance	24%	BPD 50%*; depression 30%
% treated within recommended time frame	74%*	BPD 36.7%; depression 38.3%
% attendances that were admitted	29%	BPD 31%; depression 18.5%
% attendances for injury and poisoning	27%	35%, comprising 20.8% (poison); 13.9% (injury)

* *P* < 0.001, χ^2^ analysis.

† The age range used in this comparison is not identical.

AIHW, Australian Institute of Health and Welfare; BPD, borderline personality disorder.

### Re‐presentation to ED

The majority (73%) of BPD patients attended ED more than once during the audit year (average 4.81 ± 6.63 times; range 2–78). By contrast, 42% of ‘depression only’ patients attended ED more than once, with lower average re‐presentation rates (average 1.87 ± 1.60 times; range 2–19). Review of re‐attendance during the 6 years that records were available revealed that 23 BPD‐diagnosed patients from the present study had each attended ED more than 100 times (Table 5). Cumulatively, these 23 patients had attended ED 3359 times, with an average of 146 visits per patient.

**TABLE 5:** ED attendance frequency among the 583 matched patients during the previous 6 years

ED attendance	BPD cases	Depression (no BPD) cases
More than once	541 (93%)	440 (75%)
2–19 times	393 (67%)	429 (74%)
20–100 times	125 (21%)	11 (1.9%)
More than 100 times	23 (3.9%)	0 (0%)

BPD, borderline personality disorder.

Analysis of re‐presentations of patients diagnosis with BPD within the audit year defined presentation frequency as low (once or twice; *n* = 258), moderate (three to nine times; *n* = 262) or high (10 or more times; *n* = 63). The sex and age distributions were similar across the three frequency cohorts. The likelihood of patients being under the care of a psychiatrist or case‐managed increased as a function of attendance frequency. The prevalence of substance use disorder, anxiety and bipolar affective disorder was similar across low, moderate and high ED attenders. Depressive disorder was co‐diagnosed less frequently in the high attendance cohort; diagnosis of schizophrenia/psychosis was more prevalent. Polypharmacy was greatest among high ED attenders (51%).

The high ED attendance cohort was most likely to arrive by ambulance (63.5%), receive a triage category rating of ‘2’ (imminently life threatening; 25.4%), more likely to have an ‘aggression alert’ on their medical record (30.2%) and least likely to be admitted to an inpatient unit (14.3%).

All attendance categories showed evidence of a chronic pattern of behaviour, with suicidal ideation, self‐injury and overdose accounting for most of their ED presentations. With respect to trauma, the medical records included very little information concerning psycho‐social factors precipitating ED visits. Some records contained information regarding (childhood) sexual abuse and assault, particularly for the high attendance cohort (24%).

## Discussion

The present study examined hospital emergency records over a 12‐month period to explore presentation features and management of people diagnosed with BPD. Despite the perception that people with BPD are disproportionately reliant on emergency services,^3^, ^6^ ED attendances by people previously diagnosed with BPD represented fewer than 2% of total ED presentations. The characteristics of 583 unique ED presentations clearly describe people suffering from severe BPD‐related mental health difficulties. It is possible that these low numbers – which only include people previously diagnosed with BPD – reflect a lack of documentation of the BPD diagnosis, as well as delays in formally diagnosing BPD.^16^

Of these 583 patients, 73% attended ED on more than one occasion during the audit year, averaging five occasions per person. Frequent re‐presentation may contribute to the perception that people diagnosed with BPD represent a greater proportion of ED attendees than what is reported in this study. Previous studies demonstrate the propensity for re‐presentation by people experiencing crises associated with personality disorder.^17^, ^18^

In the present study, BPD‐diagnosed patients who visited ED were predominantly young and female, most often presenting with suicidal ideation, self‐injury and/or drug overdose. This is despite similarities in community prevalence of BPD in men and women^19^, ^20^, ^21^ which may reflect systemic under‐diagnosis of men within the health system as well as a reduced likelihood of men to seek help from ED.^22^ The younger age distribution reflects the age range during which symptoms tend to be most acute; BPD expression changes as people age, with reductions in acuity and suicidality and changes in the modes of self‐injury.^23^, ^24^

When compared with a presentation‐matched depression‐only comparator group, BPD patients attended ED more frequently, experienced greater comorbidity, multi‐service involvement and were more likely to arrive by ambulance. These findings are supported by previous research, with a diagnosis of major depression associated with a lower frequency of ED presentations in comparison with personality disorder.^14^ These features, in addition to the higher prevalence of case management and inclusion of a psychiatrist in the care team, suggest that BPD‐related ED presentations are associated with more complex and severe personality disorder. The likelihood of arrival by ambulance was greatest among patients with the highest numbers of re‐presentations. It is possible that patients learn that arriving by ambulance is a faster way to get the help they need in ED, but may also reflect an escalation in severity of self‐injury, the incidence of which also rose in frequent re‐presenters. Paradoxically, inpatient admission was less likely for the most frequent presenters.

Previous studies clearly demonstrate that BPD‐specific psychotherapy reduces suicidality, self‐injury and hospitalisation.^24^, ^25^ International studies show that successful referral from ED to appropriate follow‐up treatment results in fewer ED re‐presentations,^26^, ^27^ thereby reducing reliance on ED staff and resources.^18^ Despite being case‐managed and under the care of a psychiatrist/psychologist, many patients in the present study continue to seek help in ED, suggesting that they are not receiving timely evidence‐based treatment. Unfortunately the challenges related to availability and affordability of BPD‐appropriate psychotherapeutic treatment are widely reported.^28^, ^29^

With respect to ED outcomes, 50% of patients diagnosed with BPD were admitted to inpatient units, medical units or referred to community mental health services, reflecting the severity, acuity and perceived level of risk associated with their presentation. A further 25% were referred to the care of a GP, whose professional training does not necessarily include the management or treatment of BPD. It is perhaps not surprising that 73% of the BPD‐diagnosed cohort re‐attended ED during the study period, with 11% re‐presenting 10 or more times. Anxiety around the perceived risk for patients who experience chronic suicidality may contribute to more frequent hospitalisation. However, it was notable that the lowest admission rate was associated with patients who attended ED most often, suggesting that evidence against routine hospitalisation of patients with BPD may have been influential in these decisions.^30^

Despite the ongoing lack of indication for any prescription medication in the treatment of BPD,^31^ pharmacotherapy is frequently prescribed, often as a first‐line treatment.^2^, ^32^ Polypharmacy was evident in the present study, particularly in people who attended ED on 10 or more occasions. Prescription medications were frequently implicated in overdose presentations to ED, far exceeding illicit drug or alcohol‐related overdoses, underscoring the inherent risk associated with prescribing psychoactive medications to chronically suicidal patients. An earlier study of people with BPD who died by suicide found that overdose was the cause of death in 21% of men and 40% of women.^7^

EDs conform to a medical model for delivering emergency medicine and responding to acute crises. In terms of crisis service availability, the ED is the only option for urgent medical and mental health‐related help‐seeking, particularly outside normal business hours. It is also the primary route through which people are admitted to mental health inpatient units in the public hospital system. The observation that the BPD and depression‐only cohorts both experienced longer waiting times than the national averages may reflect the poor resourcing of EDs for managing mental health‐related crisis presentations. This may also be a consequence of triage category; the majority of patients in this audit were assigned to categories 3 or 4 which typically leads to delayed treatment due to prioritisation of categories 1 and 2. The absence of information recorded in the medical records regarding trauma history and psychosocial factors precipitating ED visits suggests that psychological thinking and trauma‐informed care is not routinely available during ED visits. Asking patients ‘what is happening for you’ is a comparatively simple psychotherapeutic intervention that may improve understanding and promote empathy, de‐escalating emotionally charged situations and harmful counter‐transference with ED staff.

### Limitations

Limitations in the data collection include the seemingly arbitrary way in which information about BPD presentations was sometimes recorded, particularly with respect to nomination of the primary reason for presentation. A further limitation was the non‐specific and sometimes inaccurate recording of mental health diagnoses. The present study relied exclusively on the accurate recording of key events during ED visits, therefore omissions or inaccuracies in reporting may have affected the data analysis and interpretation.

## Conclusions

Despite the perception that people diagnosed with BPD place a heavy burden on acute crisis services, the present study found that the overall proportion of ED presentations by people previously diagnosed with BPD was around 2%. It is clear that people with BPD who come to ED likely represent the most unwell who go on to re‐present to ED on multiple occasions. The results of this audit serve as an important catalyst for developing screening, brief psychological intervention training, and effective referral strategies to help arrest crisis‐driven ED presentations of people suffering from BPD.

## References

[1] Lieb K , Zanarini MC , Schmahl C , Linehan MM , Bohus M . Borderline personality disorder. Lancet 2004; 364: 453–61. 15288745 10.1016/S0140-6736(04)16770-6

[2] Paris J . Borderline personality disorder. CMAJ 2005; 172: 1579–83. 15939918 10.1503/cmaj.045281 PMC558173

[3] Grenyer BF . An integrative relational step‐down model of care: the project air strategy for personality disorders. ACPARIAN 2014; 9: 8–13.

[4] Vandyk A , Bentz A , Bissonette S , Cater C . Why go to the emergency department? Perspectives from persons with borderline personality disorder. Int. J. Ment. Health Nurs. 2019; 28: 757–65. 30779279 10.1111/inm.12580

[5] Barrachina J , Pascual JC , Ferrer M *et al*. Axis II comorbidity in borderline personality disorder is influenced by sex, age, and clinical severity. Compr. Psychiatry 2011; 52: 725–30. 21349508 10.1016/j.comppsych.2010.11.009

[6] Jackson HJ , Burgess PM . Personality disorders in the community: a report from the Australian National Survey of Mental Health and Wellbeing. Soc. Psychiatry Psychiatr. Epidemiol. 2000; 35: 531–8. 11213842 10.1007/s001270050276

[7] Broadbear JH , Dwyer J , Bugeja L , Rao S . Coroners' investigations of suicide in Australia: the hidden toll of borderline personality disorder. J. Psychiatr. Res. 2020; 129: 241–9. 32823217 10.1016/j.jpsychires.2020.07.007

[8] Chesney E , Goodwin GM , Fazel S . Risks of all‐cause and suicide mortality in mental disorders: a meta‐review. World Psychiatry 2014; 13: 153–60. 24890068 10.1002/wps.20128 PMC4102288

[9] Ring D , Lawn S . Stigma perpetuation at the interface of mental health care: a review to compare patient and clinician perspectives of stigma and borderline personality disorder. J. Ment. Health 2019; doi: 10.1080/09638237.2019.1581337. 30862201

[10] Tusiani‐Eng P , Yeomans F . Borderline personality disorder: barriers to borderline personality disorder treatment and opportunities for advocacy. Psychiatr. Clin. North Am. 2018; 41: 695–709. 30447733 10.1016/j.psc.2018.07.006

[11] Hong V . Borderline personality disorder in the emergency department: good psychiatric management. Harv. Rev. Psychiatry 2016; 24: 357–66. 27603743 10.1097/HRP.0000000000000112

[12] Lawn S , McMahon J . Experiences of care by Australians with a diagnosis of borderline personality disorder. J. Psychiatr. Ment. Health Nurs. 2015; 22: 510–21. 26122817 10.1111/jpm.12226 PMC4755162

[13] Pasic J , Russo J , Roy‐Byrne P . High utilizers of psychiatric emergency services. Psychiatr. Serv. 2005; 56: 678–84. 15939943 10.1176/appi.ps.56.6.678

[14] Sullivan PF , Bulik CM , Forman SD , Mezzich JE . Characteristics of repeat users of a psychiatric emergency service. Psychiatr. Serv. 1993; 44: 376–80. 10.1176/ps.44.4.376 8462947

[15] Australian Institute of Health and Welfare . Emergency Department Care 2015–16: Australian Hospital Statistics. Canberra: Australian Institute of Health and Welfare, 2016.

[16] Sisti D , Segal AG , Siegel AM , Johnson R , Gunderson J . Diagnosing, disclosing, and documenting borderline personality disorder: a survey of psychiatrists' practices. J. Pers. Disord. 2016; 30: 848–56. 26623537 10.1521/pedi_2015_29_228

[17] Shaikh U , Qamar I , Jafry F *et al*. Patients with borderline personality disorder in emergency departments. Front. Psych. 2017; 8: 136. 10.3389/fpsyt.2017.00136 PMC5543278 28824467

[18] Bourke J , Murphy A , Flynn D *et al*. Borderline personality disorder: resource utilisation costs in Ireland. Ir. J. Psychol. Med. 2021; 38: 169–76. 34465404 10.1017/ipm.2018.30

[19] Torgersen S , Kringlen E , Cramer V . The prevalence of personality disorders in a community sample. Arch. Gen. Psychiatry 2001; 58: 590–6. 11386989 10.1001/archpsyc.58.6.590

[20] Grant BF , Chou SP , Goldstein RB *et al*. Prevalence, correlates, disability, and comorbidity of DSM‐IV borderline personality disorder: results from the wave 2 National Epidemiologic Survey on alcohol and related conditions. J. Clin. Psychiatry 2008; 69: 533–45. 18426259 10.4088/jcp.v69n0404 PMC2676679

[21] Tomko RL , Trull TJ , Wood PK , Sher KJ . Characteristics of borderline personality disorder in a community sample: comorbidity, treatment utilization, and general functioning. J. Pers. Disord. 2014; 28: 734–50. 25248122 10.1521/pedi_2012_26_093 PMC3864176

[22] Harris MG , Diminic S , Reavley N , Baxter A , Pirkis J , Whiteford HA . Males' mental health disadvantage: an estimation of gender‐specific changes in service utilisation for mental and substance use disorders in Australia. Aust. N. Z. J. Psychiatry 2015; 49: 821–32. 25818334 10.1177/0004867415577434

[23] Beatson J , Broadbear JH , Sivakumaran H *et al*. Missed diagnosis: the emerging crisis of borderline personality disorder in older people. Aust. N. Z. J. Psychiatry 2016; 50: 1139–45. 27056175 10.1177/0004867416640100

[24] Zanarini MC , Frankenburg FR , Hennen J , Silk KR . The longitudinal course of borderline psychopathology: 6‐year prospective follow‐up of the phenomenology of borderline personality disorder. Am. J. Psychiatry 2003; 160: 274–83. 12562573 10.1176/appi.ajp.160.2.274

[25] Warrender D , Bain H , Murray I , Kennedy C . Perspectives of crisis intervention for people diagnosed with “borderline personality disorder”: an integrative review. J. Psychiatr. Ment. Health Nurs. 2021; 28: 208–36. 32367638 10.1111/jpm.12637

[26] Prada P , Perroud N , Rüfenacht E , Nicastro R . Strategies to deal with suicide and non‐suicidal self‐injury in borderline personality disorder, the case of DBT. Front. Psychol. 2018; 9: 2595. 30619004 10.3389/fpsyg.2018.02595 PMC6304419

[27] Linehan MM , Comtois KA , Murray AM *et al*. Two‐year randomized controlled trial and follow‐up of dialectical behavior therapy vs therapy by experts for suicidal behaviors and borderline personality disorder. Arch. Gen. Psychiatry 2006; 63: 757–66. 16818865 10.1001/archpsyc.63.7.757

[28] Iliakis EA , Sonley AK , Ilagan GS *et al*. Treatment of borderline personality disorder: is supply adequate to meet public health needs? Psychiatr. Serv. 2019; 70: 772–81. 31138059 10.1176/appi.ps.201900073

[29] Carrotte E , Hartup M , Blanchard M . “It's very hard for me to say anything positive”: a qualitative investigation into borderline personality disorder treatment experiences in the Australian context. Australian Psychologist 2019; 54: 526–35.

[30] Paris J . Is hospitalization useful for suicidal patients with borderline personality disorder? J. Pers. Disord. 2004; 18: 240–7. 15237044 10.1521/pedi.18.3.240.35443

[31] Lieb K , Vollm B , Rucker G *et al*. Pharmacotherapy for borderline personality disorder: Cochrane systematic review of randomised trials. Br. J. Psychiatry 2010; 196: 4–12. 20044651 10.1192/bjp.bp.108.062984

[32] Starcevic V , Janca A . Pharmacotherapy of borderline personality disorder: replacing confusion with prudent pragmatism. Curr. Opin. Psychiatry 2018; 31: 69–73. 29028643 10.1097/YCO.0000000000000373

